# Effectiveness of a comic book intervention on mental health literacy among adolescents and youth in Burkina Faso: a randomized controlled trial protocol

**DOI:** 10.1186/s12889-025-21325-2

**Published:** 2025-01-09

**Authors:** Irene Brandt, Sylvain Some, Ourohiré Millogo, Oumar Sourabié, Jacob Burns, Sachin Shinde, Matthias Haucke, Nathan Sivewright, Christine Neumann, Shraddha Bajaria, Katian Napon, Clarisse Dah, Marina Taonda, Jason T. Siegel, Mary Mwanyika Sando, Till Bärnighausen, Ali Sié, Shuyan Liu

**Affiliations:** 1 Department of Psychiatry and Psychotherapy (Campus Charité Mitte), Charité – Universitätsmedizin Berlin, Berlin, Germany; 2 Heidelberg Institute of Global Health, Heidelberg, Germany; 3 Nouna Health Research Center (CRSN), Nouna, Burkina Faso; 4 Institut de Recherche en Sciences de La Santé, Ouagadougou, Burkina Faso; 5 Centre Hospitalier Universitaire Sourô Sanou, Bobo-Dioulasso, Burkina Faso; 6 TUM School of Medicine and Health, Technical University of Munich, Munich, Germany; 7 Department of Global Health and Population, Harvard T.H. Chan School of Public Health, Boston, MA USA; 8 Africa Academy for Public Health (AAPH), Dar es Salaam, Tanzania; 9 School of Social Science, Policy, and Evaluation, Claremont Graduate University, Claremont, CA USA; 10 Africa Health Research Institute, Somkhele and Durban, South Africa; 11 German Center for Mental Health (DZPG), Berlin and Heidelberg, Germany

**Keywords:** Mental disorder knowledge, Health education and communication, Stigma, Help-seeking, Depression and anxiety, Mental illness, Light-touch intervention, Non-specialist providers, Low-and middle-income countries, Sub-Saharan Africa

## Abstract

**Background:**

In Burkina Faso, nearly half of the population is under 15 years old, and one in four adolescents experience depression. This underscores the critical need to enhance mental health literacy among adolescents and youth, empowering them to manage their mental well-being effectively. Comic books offer an engaging approach to health education, yet their effectiveness in addressing mental health remains largely untested. Our study aims to fill this gap by evaluating the effectiveness of comic books in enhancing mental health literacy among adolescents and young adults aged 10–24 years.

**Methods:**

We will recruit 2,007 participants aged 10–24, stratify them by age, and randomly assign them to three groups (1:1:1): a comic book intervention group (Intervention 1), a text-only flyer group (Intervention 2), and a control group with no intervention. The primary outcome will be mental health literacy and secondary outcomes will include anxiety, depression, and intentions to cope.

**Discussion:**

We hypothesize that the comic book intervention and flyer intervention will prove more effective in improving mental health literacy than the control group. We further hypothesize that for younger adolescents (10–14), the comic book will be more effective at increasing mental health literacy than the flyer. Conversely, we hypothesize that the flyer will be more effective in increasing mental health literacy for older adolescents (15–24). Our study will provide evidence on novel interventions designed to enhance mental health literacy among adolescents and young adults in low-resource settings.

**Trial registration:**

This trial has been registered on the German Clinical Trials Register (DRKS), with the registration number DRKS00034242.

**Supplementary Information:**

The online version contains supplementary material available at 10.1186/s12889-025-21325-2.

## Introduction

It is estimated that one in seven adolescents experiences a mental disorder globally, yet this vulnerable group is largely undiagnosed and untreated [1]. The World Health Organization categorizes adolescents from the ages of 10 to 19 years of age and youth as 15 to 24 [2]. This mental health crisis for adolescents and youth is especially critical in low-and middle-income countries (LMICs), where the high demand for quality mental health services is often met by low supply, as seen in Sub-Saharan Africa (SSA) [3, 4]. Burkina Faso, one of seven SSA countries ranked in the lower 5th percentile out of the Human Development Index [5], exemplifies this issue. While Burkina Faso has over 20 million inhabitants, there are only eleven psychiatrists, eighty-six nurses specialized in mental health, five psychologists, and ten neurologists who are practicing in the public healthcare systems [6].

A national survey from 2015 indicated that 41.5% of Burkina Faso’s population currently suffers from at least one mental disorder, with depression being the most common [7]. The country also has a very young population, with almost half (45.3%) under 15 years old [6]. Approximately one in four adolescents aged 10 to 19 years (26.4%) in Burkina Faso reported experiencing depressive symptoms [8]. Another study surveyed Burkina Faso youth aged 12 to 20 years on the prevalence of self-injurious thoughts and behaviors and found that 8% of 12–13-year-olds reported life was not worth living, with this percentage increasing to 20% among 18–20-year-olds [9].

Burkina Faso’s 2020–2024 Mental Health Strategic Plan [6] identifies several priority areas, including conducting mental health research (Priority Area 6) and focusing on specific groups such as adolescents (Priority Area 13). Advancing research on adolescent mental health can focus on promotion, prevention, and/or treatment. The WHO recommends increasing mental health knowledge and understanding as a strategy to prevent and promote mental health issues [10]. Increasing mental health literacy (MHL) in this population represents an essential intervention pathway to understand mental health conditions, their symptoms, causes, and available treatments.

The concept of MHL is defined as “understanding how to obtain and maintain positive mental health; understanding mental disorders and their treatments; decreasing stigma related to mental disorders, and enhancing help-seeking efficacy” [11, 12]. Individuals with high MHL are better equipped to make informed decisions about mental health treatment and use effective coping strategies [13]. While adolescents and youth broadly have lower rates of MHL [14], this problem is more pronounced in LMICs [15]. Most of the studies that target MHL take place in high-income countries and depend on the availability of trained mental health professionals, such as therapists, psychiatrists, and social workers, to deliver the interventions [16]. This furthers the need for MHL interventions delivered by non-specialist health providers (NSHPs) such as teachers, lay health workers, and community health workers [17].

In resource-constrained settings it can also be important to intervene efficiently. “Light-touch” interventions are low-cost and minimally invasive interventions [18]. Light-touch interventions are typically aimed at improving psychological well-being for the subclinical population [19]. Such interventions have been demonstrated to improve mental health in low-income settings [20].

Many government organizations create brochures to educate the public on the basics of mental health education (see https://www.nimh.nih.gov/health/publications). The use of brochures to increase health literacy is a widespread practice, and there have been mixed results regarding whether a brochure is more effective than a more visually engaging delivery mechanism, such as an illustrated brochure, brochure with photographs, or video [21].

Comic books offer a low-cost, engaging approach to health education, accessible to varying literacy levels by providing pictures that provide visual explanations of the text [22]. A comic's “fun factor” of a comic draws in individuals who would otherwise be reluctant to read text-only narratives [23]. There is some evidence that comic books are especially effective for an adolescent population in communicating health knowledge [24]. However, to the best of our knowledge, there have been no randomized control trials testing the effectiveness of using a comic book to improve MHL for adolescents and youth in LMICs.

This study aims to evaluate whether an illustrated comic book or a brochure-like flyer (text-only) that describes the mental disorders of anxiety and depression as well as information around help-seeking and coping, is more effective at increasing mental health literacy for certain age groups. We hypothesize that both the comic book and the flyer will increase mental health literacy for all ages compared to a control group that receives no intervention. However, we hypothesize that the comic book will be more effective in increasing mental health literacy with younger adolescents (ages 10–14) compared to older adolescents and youth (ages 15–24) due to the engaging nature of illustrations for young readers. The primary outcome will be mental health literacy and secondary outcomes will include anxiety, depression, and intentions to cope. We will also explore how the information in the comic book and flyer might impact anxiety and depression scores as previous studies have shown that at times, individuals are negatively impacted after consuming information about depression [25]. The primary and secondary outcomes will be assessed directly following the implementation of the intervention for the comic book and flyer groups.

## Methods

### Participants and recruitment

This randomized controlled trial study is part of the research grant DASH for the design and evaluation of adolescent health interventions and policies in Sub-Saharan Africa (SSA). DASH is a network comprising public health research and training institutions from seven SSA countries (Burkina Faso, Ethiopia, Ghana, Nigeria, South Africa, Tanzania, Uganda), as well as Germany and the United States. It aims to address critical research gaps related to the need for interventions and policies and their design, evaluation, and transportability across three domains: nutrition and physical activity, sexual and reproductive health, and mental health and violence (https://dash-rhissa.org/). This trial is integrated into the initial round of longitudinal data collection of a cohort of adolescents and young adults for each country, which will hereafter be referred to as the ‘DASH cohort study’. A Health and Demographic Surveillance System (HDSS) operates in each of the study communities, meaning that the DASH cohort study can use existing sampling frames of the full population to sample and recruit participants.

Within the DASH cohort study, we will sample and recruit 2,007 adolescents and youth aged between 10 and 24 years (with equivalent proportions from the age categories 10–14; 15–19; and 20–24 years) in the Burkina Faso study community. Where the initial sample is not sufficient to reach 2,007 participants due to refusal to participate or incorrect sampling frames, we will draw further individuals from the existing sampling frame, until 2,007 participants are enrolled. These individuals will be followed up annually for four waves of data collection. This sample of 2,007 will also serve as the study sample for this trial.

## Sample size and power considerations

To estimate the sample size and conduct the power analysis, we used the *R* package “Pwr” and the statistical software G*Power [26]. We calculated the minimum detectable effect size (MDE) for primary and secondary outcomes, which represents the smallest effect size that would be sufficient to detect statistical significance, based on a predetermined level of significance (*α*), sample size (*N*), and statistical power (1—*β*). We began by computing the power for t-tests of means to achieve a target power of 0.80. This process was conducted separately for each of the two waves in our study, which include three treatment arms and three age subgroups. In our calculations, we used the guideline* α* = 1 − (1 − 0.05) ^(1/√*h*) [27], where *h* was computed based on the number of treatment arms and outcomes. The overall sample size was set at *N* = 2007. For Wave 1, we allocated the sample size as N/3 per treatment arm. Anticipating a 10% attrition rate for follow-up measurements in Wave 2, the adjusted sample size was calculated as *N* = *n* = (sample/3) * (1—attrition). For the subgroup analysis in Wave 1, the sample size per age subgroup was assumed as *N* = sample/9, (i.e., N divided by 3 treatments arms * 3 age subgroups). Similarly, the adjusted sample size for Wave 2 subgroup analysis, the adjusted sample size was *n* = (sample/9) * (1—attrition). We used *R* Power to input the calculated MDEs and illustrate how each subgroup sample size corresponds to power levels (See Table 1).

**Table 1:** Required sample size and minimum detectable effect size (MDE) for each wave and full and subgroups

Sample	Treatment (N)	Comparison (N)	Cohen’s *d* Wave 1	Cohen’s *d* Wave 2
Full treatment (Comic book, Flyer, Control)	669	669	0.17	0.18
Age sub-groups	223	223	0.32	0.34

There is a lack of studies investigating the impact of comic book intervention on mental health literacy in Africa. To ensure that the calculated effect estimate is something that we could reasonably expect, we compare our calculated effect sizes with those from a related study in a non-mental health domain. Shin and colleagues (2022) investigated the effectiveness of a comic book intervention in East Africa on knowledge about human papillomavirus (HPV) types [28]. They reported that the mean percentage of correctly answered questions about HPV pre-intervention was 44%, which increased to 82.9% post-intervention. The study included a sample size of *n* = 64 for the pre-test and *n* = 72 for the post-test assessments. Cohen's d effect size was calculated using the following formulas.

$$d=\frac{Mean1-Mean2}{PooledStandardDeviation}$$

$$PooledSD=\sqrt{\frac{\left(n1-1\right)*{SD}^{2}1+\left(n2-1\right)*{SD}^{2}2}{n1+n2-2}}$$

This resulted in Cohen’s *d* = 3.58, which indicates a large effect size.

In addition, there was a previous study on age differences in mental health literacy [19] that used Cohen’s *h* values, a measure similar to Cohen’s *d* but applicable to differences between proportions or probabilities. A difference with *h* = 0.2 is considered “small”, *h* = 0.5 indicates a “medium” difference, and *h* = 0.8 represents a “large” difference. Their findings revealed that adults aged 70 years exhibited lower accuracy in identifying depression symptoms compared to those aged 18–24 years (Cohen’s *h* = 0.64), 25–39 years (*h* = 0.61), 40–54 years (*h* = 0.53), and 55–69 years (*h* = 0.50). Based on this, we assume a medium effect size for the impact of age on mental health literacy. In summary, our sample size is likely adequate for detecting both large and medium effect sizes, as observed in previous studies.

## Procedure

**Fig. 1:**
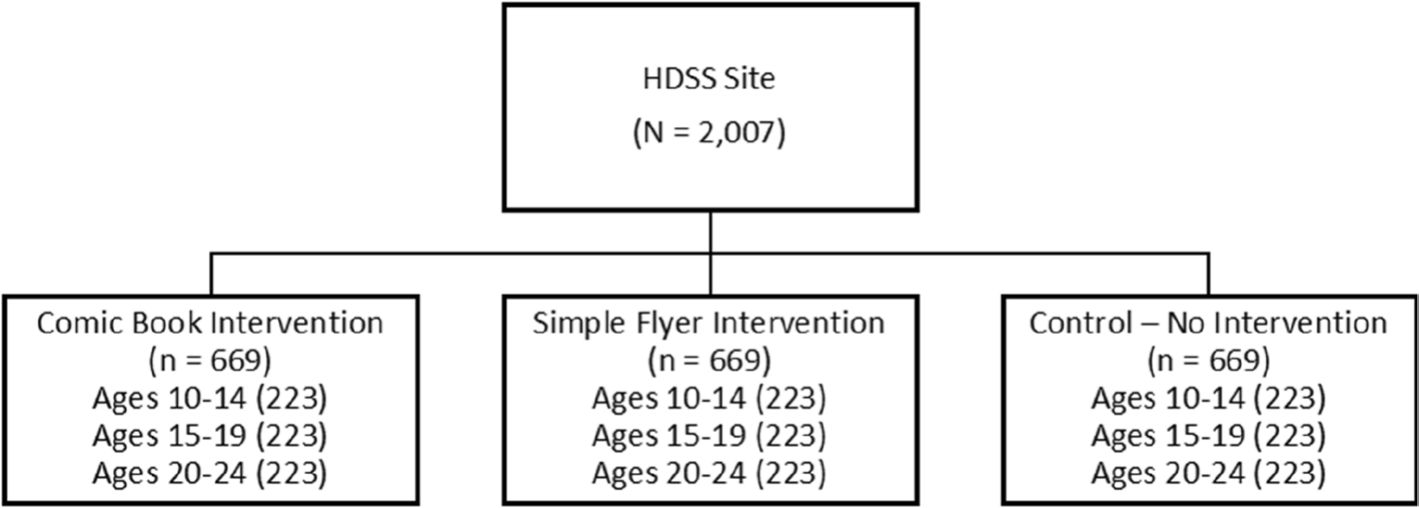
Trial flow chart

In this randomized controlled trial, each participant will be randomly assigned (1:1:1) to receive either a comic book (Intervention 1, 15 min long) a flyer (Intervention 2, 15 min long), or no intervention (Control). The randomization process will be stratified for the age category (i.e., 10–14 years; 15–19; 20–24) to ensure that within each category, a similar number is allocated to each arm (see Fig. 1). The allocation sequence will be generated by colleagues at the Technical University of Munich, who are not further involved in the conduct of the trial. They will use statistical computing software to define a random allocation sequence for all potentially eligible participants included in the pre-defined sample roster.

As part of the DASH cohort study, which this trial is nested, all participants will take part in a survey interview of approximately 60–90 min. Each interview will begin with the data collector providing the consent form. If the participant consents, they will be asked survey questions regarding mental health literacy, anxiety, and depression before the intervention is administered, which will serve as baseline measures. For participants assigned to the comic book intervention, the data collector will provide the printed comic book to the participants, reading the text aloud. For participants assigned to the flyer intervention, the process will be the same where the data collector will provide the printed flyer, reading the text out loud. Participants in the control group will be provided with no additional instructions or interventions. Participants will then be asked other survey questions regarding sexual and reproductive health providing a brief break between the intervention implementation and the post-intervention survey. All participants will be surveyed on mental health literacy, anxiety, depression, and intentions to cope as a post measure.

## Interventions

### Comic book

**Fig. 2:**
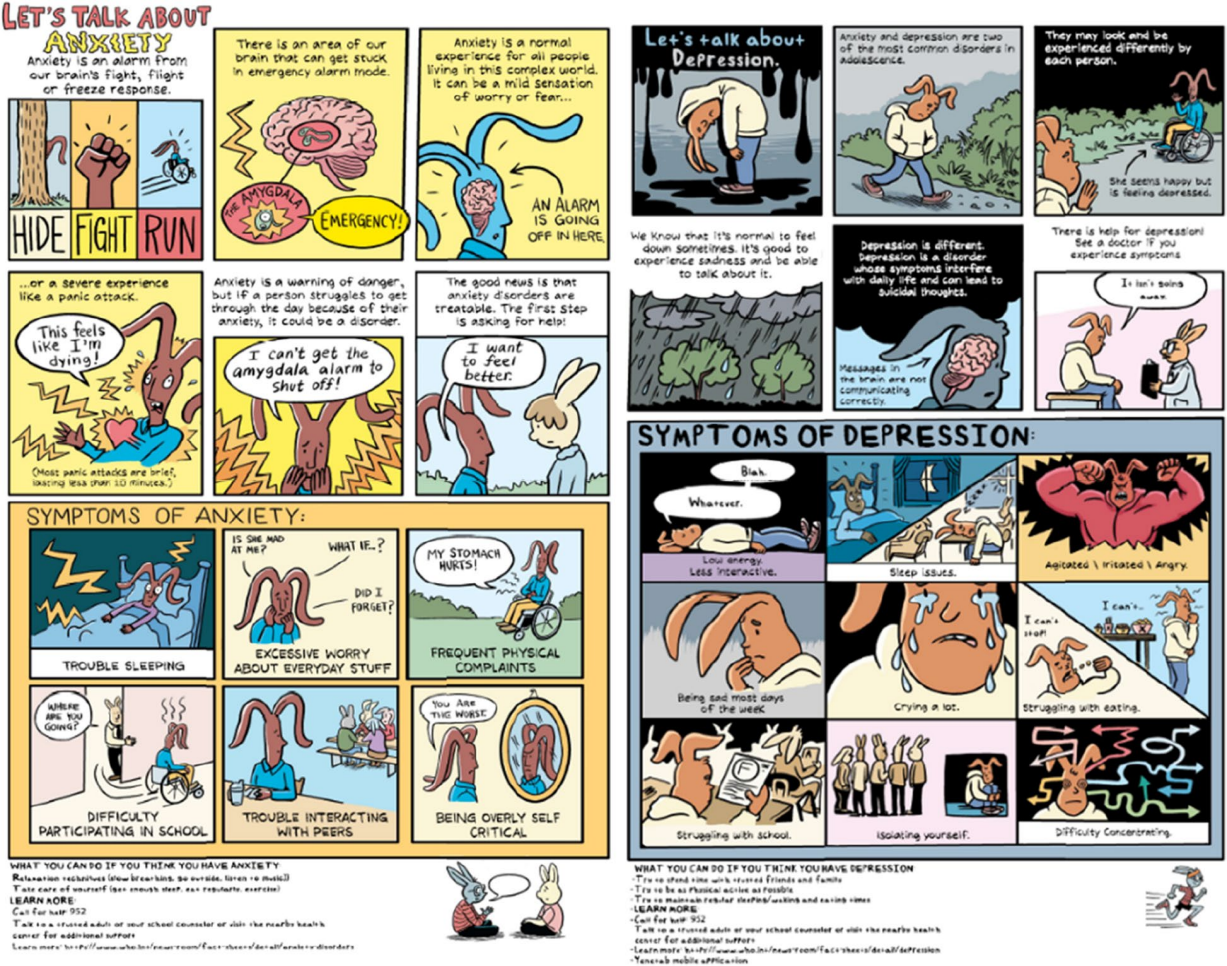
Comic book as a light-touch intervention

The original comic book titled “Let’s Talk About It” is a 28-page Graphic Guide to Mental Health that was originally co-created by the Cartoon Studies Lab for the Ohio State Department of Health (USA), specifically designed for middle and high school students (https://www.cartoonstudies.org/css-studio/cartooningprojects/mentalhealth/). As a light-touch intervention, we chose two pages from the comic book that specifically highlight the two most prevalent mental disorders in this population: Anxiety and Depression (see Fig. 2). These pages primarily convey information about mental health, understanding mental disorders, guidance on seeking help, and addressing stigma through storytelling with an illustrated cast of rabbit characters. Each page briefly explains how biological processes in the brain result in anxiety and depression and how a disorder differs from feeling stressed or feeling blue. It also covers the Diagnostic and Statistical Manual of Mental Disorders, Fifth Edition (DSM-5) [29] based on symptomology for each disorder. Notably, we adapted the rabbit character by using a darker shade of brown fur to reflect a representative skin color that is prevalent within the cultural context. Also, we collaborated closely with the local mental health expert practicing in Burkina Faso, to translate and validate the English text into French, ensuring it was culturally sensitive and appropriate. Furthermore, we integrated local information platforms at the bottom of the page to provide participants with additional resources for further learning.

### Flyer

**Fig. 3:**
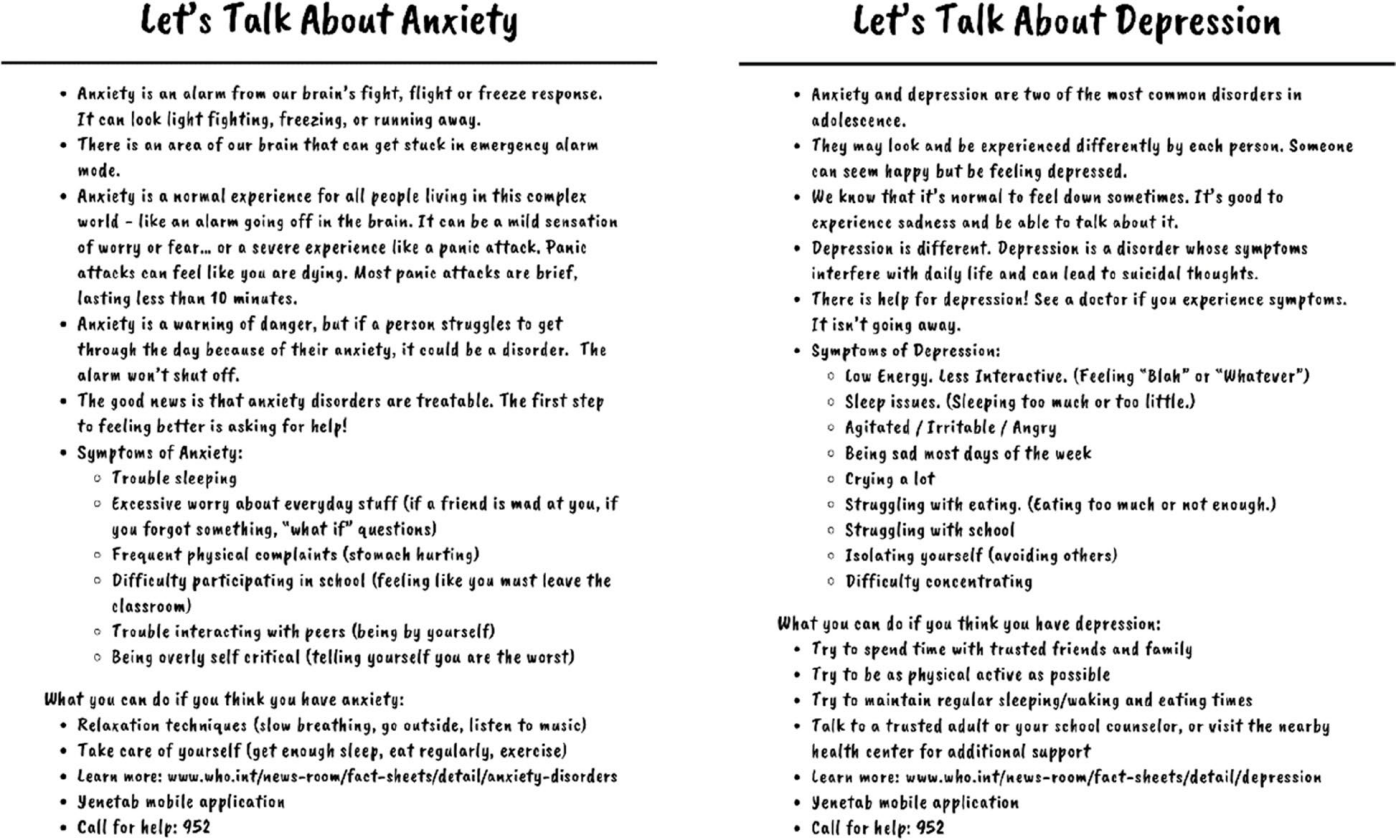
The flyer intervention

The flyer intervention is designed to resemble the commonly used health information material in public health initiatives. The flyer contains text identical to that of the comic book. However, where text within illustrations could be confusing without accompanying visuals, we adjusted the wording slightly to maintain clarity. For example, in the comic book's text accompanying the first picture for Anxiety states: “Anxiety is an alarm from our brain’s fight, flight or freeze response. Hide. Fight. Run,” which may not fully convey its meaning without the illustration. The flyer version reads: “Anxiety is an alarm from our brain’s fight, flight or freeze response. It can look like fighting, freezing, or running away.” (See Fig. 3). This adaptation ensures that the information remains accessible and understandable without accompanying visuals, maintaining the goal of the flyer as an educational tool while nevertheless remaining very close to the comic book text.

## Outcome measures

Our primary outcome will be the sum score on a revised version of a Universal Mental Health Literacy Scale for Adolescents (UMHL-A) [30], measured both before and after the intervention in wave 1 and a year later in wave 2. This scale assesses mental health literacy across domains such as mental health knowledge, mental disorder knowledge, help-seeking behavior, and stigma. The revised UMHL-A consists of 10 items, each rated on a 5-point Likert scale (1–5 points), yielding a total score ranging from of 10 to 50. Higher scores indicate a greater level of mental health literacy. Our secondary outcomes will include the total scores on the 2-item Patient Health Questionnaire (PHQ-2) [31, 32], the 2-item Generalized Anxiety Disorder (GAD-2) [32, 33], and the 2-item Intentions to Cope scale [34], all measured post-intervention.

Outcome assessment will happen as part of waves 1 and 2 of the DASH cohort study; as part of the DASH cohort study, an attempt will be made to identify and follow-up all participants from wave 1 in wave 2. Outcomes will be assessed during the survey interview conducted by a data collector. All data collectors will be trained in administering the full survey, which should ensure outcomes are collected for all participants. All scales used in our study will be translated into French and reviewed by local mental health experts for cultural sensitivity. To assess reliability, we will calculate Cronbach’s alpha within our sample [35].

First level of data entry and storage will be on a tablet before forms can be uploaded online. At this level only data collectors involved in the interview and/or data manager will have access to the data collected. Data collectors are responsible for privacy and confidentiality of the data on the device. Second level of data storage will be on the online server. Application developers will be responsible for setting up the server for online data storage. Server setup will include password protected access and different levels of access to ensure only authorized individuals can access the data online and only access their allowable sections of the application and/or data sources.

Data cleaning will first be done on the tablet before the forms are uploaded online. Data collectors will be responsible for checking sections of the questionnaire for errors. The second level of data cleaning will be done by data managers after downloading the data from the online server. At this step, data will be checked for any duplicates (using household identifiers) and any failed logical checks. Any data errors resulting from how the questions are setup and/or asked will be communicated to program managers immediately.

## Data analysis

We will assess participants’ sociodemographic characteristics, including age, gender, education levels, ethnicity, religion, household characteristics, and their parents’ highest level of education and occupation with outcome measures. To assess the effectiveness of an intervention on mental health literacy (MHL), we will build a multiple regression model. We will also assess each sub-scale component of MHL (knowledge about mental health, knowledge about mental health, help-seeking, and stigma). Here, the independent variable is “3 arms”, representing different intervention groups, and the outcome will be “MHL after the intervention.” We will control for baseline MHL scores by including them as a covariate, following the recommendation by Senn (2006) to adjust for potential baseline differences [36]. For the analysis, we will employ dummy coding for the three trial arms to compare each intervention arm against the control arm. In addition, covariates such as depressive and anxiety symptoms, intentions to cope, age, gender, and education levels will be included in the model. Secondary outcomes will be examined using three separate linear regression models, with the trial arm” treated as a factor and the control arm serving as the reference category for each secondary outcome. To ensure the validity of our multiple regression analysis, we will calculate variance inflation factor values to check for multicollinearity among the independent variables. We will conduct statistical analyses using *R version 4.1.0* (www.r-project.org).

The final cleaned, de-identified and locked dataset of the trial will be accessible by the DASH network partners. Public access to the data will be made available upon review of the request and approval by each institute’s Principal Investigators.

## Discussion

This study will contribute valuable information on light-touch interventions for improving mental health outcomes for adolescents and youth in LMICs. We will use an efficient, low cost, scalable, and validated intervention to enhance MHL among adolescents and youth in Burkina Faso. Our findings will add knowledge on the effectiveness of using a comic book to increase knowledge about mental health and mental disorders, facilitate help-seeking behaviors, reduce stigma, and thereby enhance overall MHL. In addition, we will determine if comic books are more effective for specific age groups of adolescents and youth compared to a text-only flyer. This work will advance Burkina Faso’s 2020–2024 Mental Health Strategic Plan and employ strategies suggested by the WHO Mental Health Action Plan 2013—2020. Increasing MHL has the potential to improve mental health outcomes as low levels of MHL have been linked to adverse mental health outcomes [37]. As randomized control trial studies on the effectiveness of comic books in impacting mental health literacy are few, this study will also add high quality research to future researchers who are considering this method of delivery.

Due to the nature of this light-touch intervention, its long-term effectiveness may be limited. In addition, the exposure to the light-touch intervention would benefit from repeated exposure over a period of time. By harnessing the potential of the comic book approach and incorporating lessons learned from this study, we aim to create a comprehensive comic intervention, including a digital format, in the future. Moreover, we will investigate the effect of our comic intervention on other countries part of the DASH study, including Ethiopia, Ghana, Nigeria, Uganda, South Africa, and Tanzania. Our study will provide valuable insights into innovative and engaging ways of communicating mental health information to adolescents and youth through NSHP delivery agents.

## Supplementary Information

Supplementary Material 1.

## Data Availability

No datasets were generated or analysed during the current study.
